# Trust in Vaccines: Why It Takes More than Good Faith

**DOI:** 10.3390/vaccines1030343

**Published:** 2013-08-12

**Authors:** Norman Begg

**Affiliations:** Chief Medical Officer, GlaxoSmithKline Vaccines, Avenue Fleming, 1300 Wavre, Belgium; E-Mail: norman.t.begg@gsk.com; Tel.: +32-108-55416; Fax: +32-265-63220

This *Vaccines* issue on “Confidence in Vaccines” provides sound evidence through multiple perspectives of life-saving impacts when vaccination programs are effectively implemented in a population. Yet there remain challenges to achieving this impact, including scientific, medical, manufacturing, policy-related and logistical issues. Additionally, socio-cultural, religious and political agendas can come into play, taking public health hostage and sometimes allowing the circulation of myths regarding vaccination. All of these challenges play a role in public confidence in vaccines and vaccination. What we trust, we embrace. What we do not trust, we do not embrace.

As a leading vaccine manufacturer, GlaxoSmithKline (GSK) asks: “How do we ensure that we as industry are doing our part in building and maintaining trust in vaccination?” 

Confronted with a myriad of messages in a world of heightened complexity and high-speed communications, the public is faced with the paradox of having, at the same time, too much and too little information from which to draw informed opinions. The potential vaccinee wants an answer to a basic question: “*Do those involved in bringing me (my child) this vaccine have my best interests at heart?*”

To provide that reassurance, all industry and vaccination players must examine their own ethical behaviours. There is adequate evidence that medical societies, research associations, industry and governments have reflected on standards of ethical behaviour in performing research, in assessing the safety, quality and efficacy of new products, and in defining appropriate ways of working between the medical community and the pharmaceutical industry. Virtually every organisation engaged in vaccine research, development and delivery has, or is developing, a Code of Conduct/Ethics [1,2,3,4,5,6]. At GSK, we recognise the scrutiny to which we are subject given the potential for conflict of interest between commercial objectives and our role as a partner in advancing science through research and development. We firmly hold that there is never a benefit to compromising on the level of quality in vaccine research, development and manufacturing. We know too that it is not enough to state our deeply-held values of “Patient-focus”, “Transparency”, “Integrity” and “Respect for People”; we must demonstrate these in all we do, in our corporate behaviours and in our individual activities as company representatives. To enact our mission and enable trust, it is key to play our role as scientists and physicians and to express science in a manner that is objective, accurate and complete. We define *Scientific Engagement* as the interaction and exchange of information between GSK and external communities in order to advance scientific and medical understanding, including the appropriate use of our medicines and vaccines, the management of disease and patient care.

Our approach to *Scientific Engagement* is based on five principles for application to our working practices as scientists and physicians, with the aim of earning trust and building confidence in industry:

1. *Scientific Engagement with external communities is fundamental to the progress of medical science and to meeting the needs of patients and public health.* This principle reminds us of the essential need for partnership with external experts with complementary capabilities, in order to understand healthcare needs and environments, and to advance science and deliver vaccines more rapidly. Interactions between industry and clinicians who acted as clinical trial investigators have facilitated clinical research resulting in registration of new vaccines. Also, a new model of public–private partnerships enables the development of vaccines which may not otherwise be sustainable commercially and demonstrates how external interactions not only promote the advancement of science but can also unblock barriers to new products for diseases of the developing world.
2. *GSK physicians and scientists engage in the highest standards of peer-to-peer scientific dialogue to increase understanding of diseases and develop effective prevention and treatment therapies.* Industry scientists and physicians come from a vast range of clinical, academic and government institutional experiences and settings. Even with the available staff expertise, research within the company must be subject to challenge and validation by the external community and, as stated in the second principle, these interactions should be of the highest standards in science. Mutual respect builds as shared knowledge and intellectual challenge enables researchers and physicians to advance science for the benefit of health; this industrial science credibility is a cornerstone to building public confidence. Working practices adopted by GSK that realise this principle include the selection of appropriate venues for scientific interactions, involvement of staff members that have the scientific knowledge to engage productively with peers, transparency in how we pay for services from medical consultants, and refraining from practices that could contribute to real or perceived misconduct in external interactions.
3. *Scientific Engagement is driven by legitimate scientific need. It is balanced, appropriate and proportionate to the scientific need and intent. Scientific Engagement activities or behaviours cannot be, or be perceived to be, promotional or otherwise designed to influence the prescription, supply, sale or use of GSK products.* Every scientist who has ever become passionate about a research project is warned about the potential for loss of objectivity that can accompany that passion. Principle 3 calls on staff to return to scientific and medical need as the baseline from which science must be considered at all times, and to interact and communicate in a manner that is proportionate to that need. We must learn from physicians and scientists, and provide them with appropriate information through appropriate channels in a non-promotional manner. An example relates to the appropriate conduct of advisory boards involving external experts: these are run only when the need for advice is well-defined, the advice has not previously been sought and the company is unable to obtain that advice through internal expert interactions. Such advisory boards will also involve only those staff members needed to contribute to the dialogue to obtain the needed advice, facilitating open discourse; they should never be used as an opportunity to promote for commercial gain.
4. *GSK abides by external regulations and internal Policies. Our intentions and actions are driven by our values of patient focus, transparency, respect and integrity.* By the company being extremely explicit with staff via this fourth principle, employees are anchored in core values, from which every question and decision can be benchmarked.
5. *Scientific Engagement starts in the early stages of development and continues throughout the life cycle of the product and includes all areas of scientific endeavour undertaken by GSK. Accountability and authorisation for Scientific Engagement resides within the Medical Governance framework at GSK.* We work in an integrated manner across research, development, manufacturing, medical, regulatory and commercial functions. All are involved in defining strategic directions and operational plans. What this fifth principle tells staff is that accountability in the areas of medical and scientific engagement will sit under a framework of policies and procedures that reinforce our commitment to medical and scientific principles.

Of course, much more than scientific integrity comes into play to build trust in vaccine manufacturers. Industry Codes of Practice (e.g., *EFPIA Code of Practice on the promotion of prescription-only medicines to, and interactions with, healthcare professionals, PhRMA Code on Interactions with Healthcare Professionals, ABPI Code of Practice for the Pharmaceutical Industry*) set standards for the best possible practices in the promotion and advertising of medicines. We acknowledge again that words captured in Codes can only come to life through the actions and behaviours of each company employee. Industry standards need to be visible in all ways we engage with the vaccine community, in the way we promote our products, manage safety issues, and interact with regulatory bodies, Ministries of Health and customers—essentially, in all we do. 

All of this takes leadership, communication and appropriate consequences when standards are breached. 

Leadership will be taken by industry players becoming actively engaged in defending and promoting the practices that bring credibility to industry. This may mean having more open discussions regarding changes in the ways in which industry and its partners interact. Any practices that result in an expectation, implicit or explicit, of reciprocity can impact the integrity of the dialogue and particularly the perception of impropriety. Long-passed practices of offering t-shirts and gimmicks at industry booths at medical congresses gave a poor impression of the nature of the relationship between industry and healthcare professionals. This is one small example, yet the principle of integrity and transparency in all interactions can apply in many other major and minor ways.

In the article, *Sustaining Vaccine Confidence in the 21st Century*, GSK authors conclude by highlighting the critical importance of communication and collaboration between all parties—researchers, public health organisations, manufacturers, funding bodies, and more—in this vast network that enables vaccination programmes to succeed. 

Industry efforts—no matter how professionally or passionately undertaken—if uncoordinated and unaligned are sure to result in inefficiencies (manufacturing capacities that do not meet a governmental programme need), gaps in communication (safety information that is not well enough understood by the medical community to address patient questions; myths regarding vaccines’ adverse events are allowed to circulate), and lost opportunities to deliver vaccination (supply shortages). To enable greater collaboration, industry and all those involved in vaccine delivery must increase our competencies in understanding one another—defining vaccination objectives in terms of health needs, and aligning more cohesively industry deliverables to interface with public health needs in a manner that is transparent to all stakeholders and the public.

Industry and public health authorities need closer collaboration, to provide accurate and reader-friendly information that appropriately communicates the value and the safety, including risks, of vaccination. Mark Twain is quoted as saying: “A lie can travel halfway round the world while the truth is putting on its shoes.” In a world of “tweets” and viral news through social media, our communication challenge to deliver sound scientific and medical information has never been greater, so that the public can understand vaccination issues as they arise. This means new ways of communicating, which can only be defined in partnership between health authorities, healthcare professionals, and industry. We must consider if we have, today, the proper fora to advance this communication agenda. Clearly, this is an area needing more reflection and action.

All of our collaborations, partnerships and interfaces will be judged by the value delivered and the trust engendered. Independent physicians and physician organisations are speaking up about the critical importance of this collaborative spirit based on high standards of interaction between industry and the scientific community [7]. Interactions will remain subject to scrutiny, which in itself is good. It will take all members of the broad coalition of contributors to vaccine advocacy to prove that trust is merited. We must be confident in challenging one another to mutually and continuously keep the bar high. 

When breaches are encountered, industry has to be proactive and transparent in addressing any short-comings in meeting standards, requirements or legislation through open dialogue, agreed plans to address issues, and timely implementation of corrective action.

Having worked with vaccine experts and advocates in industry, governments and academia over the past 30 years, I believe we all share a common objective grounded in public health, and that our ability to deliver to that objective is clearly linked to our ability to create, learn and problem-solve together. 

We have an incredible opportunity to contribute to the advancement of world health through vaccination to improve and protect quality of life, and it will be through our combined efforts that we will achieve this goal. 

“*Do those involved in bringing me (my child) this vaccine have my best interests at heart?*” Industry leadership, communication and collaboration according to highest standards can contribute significantly to making the answer a resounding “*Yes*”.
